# Selenium-binding lactoferrin is taken into corneal epithelial cells by a receptor and prevents corneal damage in dry eye model animals

**DOI:** 10.1038/srep36903

**Published:** 2016-11-11

**Authors:** Akihiro Higuchi, Hiroyoshi Inoue, Yoshio Kaneko, Erina Oonishi, Kazuo Tsubota

**Affiliations:** 1 Research Promotion Institute, Oita University, Oita, Japan; 2 Department of Ophthalmology, Keio University, School of Medicine, Tokyo, Japan; 3 Department of Chemistry, Keio University, School of Medicine, Kanagawa, Japan; 4 Tokyo New Drug Research Laboratories, Kowa Co., Ltd., Tokyo, Japan

## Abstract

The ocular surface is strongly affected by oxidative stress, which causes many ocular diseases including dry eye. Previously, we showed that selenium compounds, e.g., selenoprotein P and Se-lactoferrin, were candidates for treatment of dry eye. This paper shows the efficacy of Se-lactoferrin for the treatment of dry eye compared with Diquas as a control drug using two dry eye models and incorporation of lactoferrin into corneal epithelial cells via lactoferrin receptors. We show the efficacy of Se-lactoferrin eye drops in the tobacco smoke exposure rat dry eye model and short-term rabbit dry eye model, although Diquas eye drops were only effective in the short-term rabbit dry eye model. These results indicate that Se-lactoferrin was useful in the oxidative stress-causing dry eye model. Se-lactoferrin was taken into corneal epithelium cells via lactoferrin receptors. We identified LRP1 as the lactoferrin receptor in the corneal epithelium involved in lactoferrin uptake. Se-lactoferrin eye drops did not irritate the ocular surface of rabbits. Se-lactoferrin was an excellent candidate for treatment of dry eye, reducing oxidative stress by a novel mechanism.

The ocular surface is strongly affected by oxidative stress caused by various factors such as light exposure including ultraviolet (UV) irradiation^1^ and chemical compounds^2^. To protect the corneal epithelium from these factors, antioxidative and xenobiotic enzymes, *e*.*g*., glutathione peroxidase (GPx), superoxide dismutase, and cytochrome P450s (CYP), are expressed in corneal epithelial cells^2^,^3^. GPx is distributed in many tissues of the body including the ocular surface^3^,^4^. GPx participates in the reduction of hydrogen peroxide and lipoperoxide^5^,^6^; therefore, the physiological role of GPx is regulation of oxidative stress. GPx contains selenium, which is essential for the enzyme activity of GPx. Selenium is an essential trace element in animals. Selenium is a component of the amino acid selenocysteine (Sec), which is a cysteine analogue with a selenium atom replacing a sulfur atom. Proteins containing Sec are called selenoproteins. Twenty-five selenoprotein genes are present in the human genome^7^. Selenium is transported from the liver to peripheral tissues by the plasma glycoprotein selenoprotein P (SeP)^8^, which is also present in extracellular fluids such as plasma^9^ and milk^10^.

Our previous study showed that SeP was also secreted in tear fluid to supply selenium to the corneal epithelium, and the concentration of SeP in tear fluid was reduced in dry eye patients^11^. The shortage of selenium from the lacrimal glands caused oxidative stress in the corneal epithelium, resulting in corneal damage. SeP eye drops prevented this corneal damage, and we concluded that SeP was useful for the treatment of dry eye^11^. Although SeP was a good candidate for clinical application for the treatment of dry eye, it is difficult to synthesize large amounts of SeP using cultured systems. Therefore, we investigated new compounds that can supply selenium to the corneal epithelium.

Tear fluid contains many kinds of anti-oxidative stress compounds such as vitamin C, glutathione, superoxide dismutase, and lactoferrin^12^,^13^. Lactoferrin also protects the corneal epithelium against UV irradiation^14^. Previous studies demonstrated that the concentration of lacrimal lactoferrin was reduced^15^ and oral administration of lactoferrin improved symptoms in dry eye patients with Sjogren’s syndrome^16^.

Lactoferrin is an iron-binding glycoprotein and is found in most exocrine fluids such as saliva, bile, pancreatic fluid, amniotic fluid and tears^17^. The most common metal ion associated with lactoferrin *in vivo* is iron in its ferric (III) form. Lactoferrin can also bind other metal ions such as copper and magnesium. We previously reported that we were successful in preparing selenium-binding lactoferrin (Se-lactoferrin)^18^. Se-lactoferrin could supply selenium to the corneal epithelium and Se-lactoferrin eye drops prevented corneal damage by reducing oxidative stress in the corneal epithelium in dry eye model rats^18^.

Se-lactoferrin could transfer selenium to the corneal epithelium, which meant that lactoferrin was incorporated into corneal epithelial cells. It is known that lactoferrin is taken into cells via lactoferrin receptors^19^,^20^. Some lactoferrin receptors have been identified including low density lipoprotein receptor-related protein 1 (LRP1) and intelectin-1. Although lactoferrin receptors were shown to be distributed in several tissues including the brain, liver, and intestine^20^,^21^,^22^, lactoferrin receptors have not yet been identified in corneal epithelial cells. Furthermore, there are few reports on quantitative analysis of uptake of lactoferrin.

In this study, we investigated the efficacy of Se-lactoferrin for the treatment of dry eye compared with 3% diquafosol tetrasodium ophthalmic solution (Diquas^®^ ophthalmic solution 3%, Diquas) as a control drug in two dry eye models. These models were not used in our previous study^18^. Diquas is an ethical drug for the treatment of dry eye. We also investigated lactoferrin incorporation into corneal epithelial cells via lactoferrin receptors.

## Results

### Evaluation of the effect of Se-lactoferrin on dry eye using the smoking rat dry eye model

**Figure 1:**
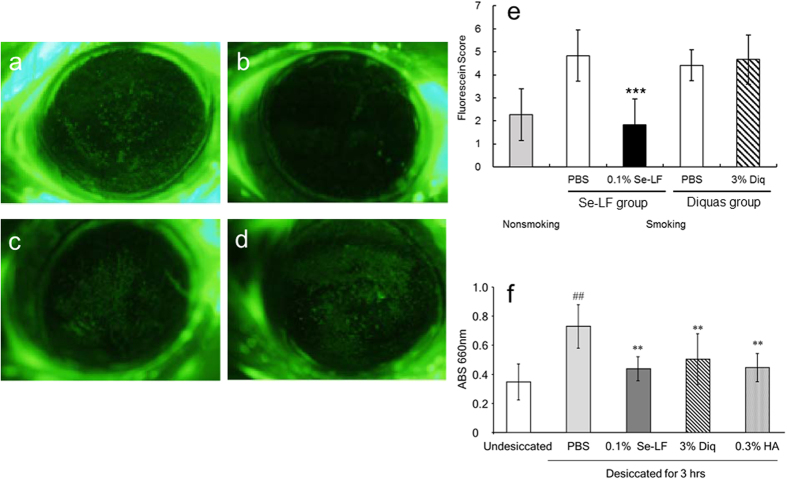
Effect of Se-lactoferrin on corneal damage in dry eye model. (**a**–**e**) Effect of Se-lactoferrin on corneal erosion in the TS exposure dry eye rat model. (**a**–**d**) Photographs of cornea stained by fluorescein after treatment with eye drops for 5 days. (**a**) PBS treatment in Se-lactoferrin group. (**b**) Se-lactoferrin eye drops in Se-lactoferrin group. (**c**) PBS treatment in Diquas group. (**d**) Diquas eye drops in Diquas group. (**e**) Fluorescein score of cornea in the eyes of dry eye rats treated with 0.1% Se-lactoferrin (Se-LF), 3% Diquas (Diq), or PBS. Results are expressed as mean ± S.D. Dunnett’s test was used to determine the significance of differences. ***Indicates a significant difference from the result in PBS treatment of the same group, P < 0.005. f: Effect of Se-lactoferrin on corneal disorder in the short-term dry eye model in rabbits. The eyes of rabbits were held open with an eyelid speculum to desiccate them. Rabbits were treated with PBS, 0.1% Se-lactoferrin (Se-LF), 3% Diquas (Diq), or 0.3% Hyalein (HA). Undesiccated eyes were eyes that were not held open by an eyelid speculum and received no eye drop treatments. All data are presented as mean ± S. D. (n = 9–10). ^##^P < 0.01, compared with the undesiccated eyes (Student’s t-test) **P < 0.01, compared with the PBS-treated eyes (Dunnett’s test).

Figure 1 shows the effect of administering Se-lactoferrin eye drops in rats with dry eye caused by TS exposure. In PBS-treated eyes, TS exposure caused an increase in the area of fluorescein staining compared with the unexposed eyes, which indicates corneal erosion (Fig. 1b). 0.1% Se-lactoferrin eye drops could suppress the increase in the area of fluorescein staining (Fig. 1a) compared with PBS treatment (Fig. 1b). On the other hand, 3% Diquas eye drops did not suppress the increase in the area of fluorescein staining (Fig. 1c,d). The fluorescein score was significantly lower in Se-lactoferrin-treated eyes than in PBS-treated eyes (Fig. 1e).

### Evaluation of the effect of Se-lactoferrin on the rabbit short-term dry eye model

The effect of Se-lactoferrin on corneal epithelium disorder was examined using another dry eye model, i.e., the rabbit short-term dry eye model (Fig. 1f). Desiccation for 3 hours induced a significant increase in methylene blue permeation compared with that in undesiccated eyes. Se-lactoferrin caused a significant reduction in corneal permeation compared with vehicle treatment. Similar reductions were observed upon treatment with 3% Diquas and 0.3% Hyalein.

### Ocular irritation of Se-lactoferrin

We studied whether repeated instillation of Se-lactoferrin induced ocular irritation in rabbits (Table 1). Upon instillation of 0.1, 0.5, or 1.0% of Se-lactoferrin, the irritation score was 1.0, 1.0, and 2.0, respectively. The main symptoms were hyperemia and secretion, but the degrees were slight. Furthermore, upon treatment with 0.1, 0.5, or 1.0% of Se-lactoferrin, the mean count of eye blinks over a 1-min period was 0.8, 0.5, and 0.8, respectively. These data suggest that Se-lactoferrin did not cause ocular irritation.

### Expression of lactoferrin receptor in corneal epithelium

Since lactoferrin was taken into cells via a lactoferrin receptor, we tried to identify the lactoferrin receptors in corneal epithelial cells. Table 2 shows the expression of lactoferrin receptors in the rat cornea. LRP1 was expressed relatively strongly (8,752-fold compared with intelectin-1), and intelectin-1 was slightly expressed in the cornea. Because similar results were obtained from the experiments using CEPI cells (Table 3), it was concluded that LRP1 is the primary lactoferrin receptor in corneal epithelium.

**Figure 2:**
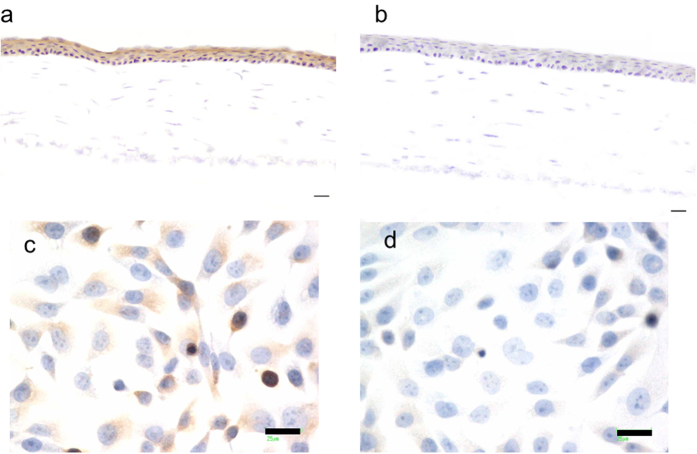
Immunohistochemical analyses of lactoferrin receptor LRP1. (**a**,**b**) Immunohistochemical staining of LRP1 in rat cornea was performed using anti-LRP1 rabbit antibody (**a**) and normal rabbit IgG (**b**). Scale bar, 20 μm. Original magnification: ×20. (**c**,**d**) Immunohistochemical staining of LRP1 in CEPI cells was performed using anti-LRP1 rabbit antibody (**c**) and normal rabbit IgG (**d**). Scale bar, 25 μm. Original magnification: ×40.

To confirm LRP1 expression in the cornea, immunohistochemical analysis was performed using anti-LRP1 antibody. LRP1 was stained in corneal epithelium by using anti-LRP1 antibody (Fig. 2a), but was not stained by using normal rabbit antibody (Fig. 2b). LRP1 was also stained in CEPI cells by using anti-LRP1 antibody (Fig. 2c). It was revealed that LRP1 was localized at the corneal epithelium.

### Lactoferrin uptake into corneal epithelial cells

**Figure 3:**
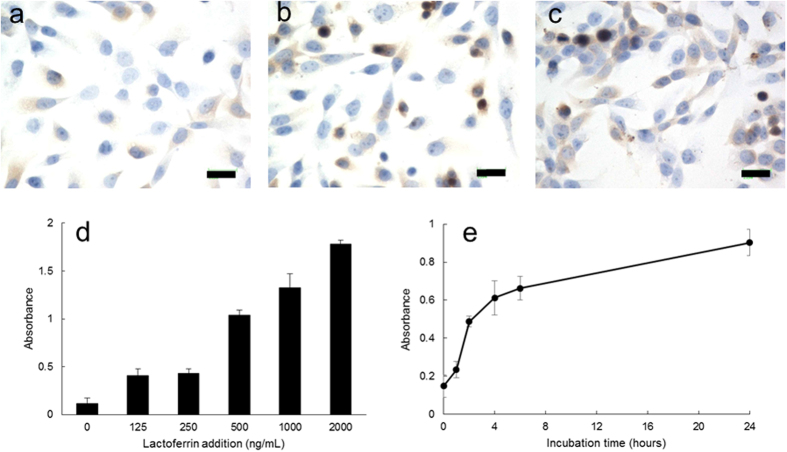
Lactoferrin uptake into corneal epithelial cells. CEPI cells were incubated in the presence or absence of lactoferrin. (**a**–**c**) Immunohistochemical staining of lactoferrin in CEPI cells was performed using anti-lactoferrin rabbit antibody. CEPI cells were incubated in the presence of 0 (**a**), 0.1 (**b**), or 0.5 (**c**) mg/mL lactoferrin. Scale bar, 25 μm. Original magnification: ×40. (**d**,**e**) Lactoferrin intake into CEPI cells was assayed by ELISA. (**d**) CEPI cells were incubated in the presence of 0, 125, 250, 500, 1000, or 2000 ng/mL lactoferrin for 72 hours. Results are expressed as mean ± S.D. (n = 5). (**e**) CEPI cells were incubated in the presence of 500 ng/mL lactoferrin for 0, 1, 2, 4, 6, or 24 hours. Results are expressed as mean ± S.D. (n = 6).

Lactoferrin uptake was visualized by using anti-lactoferrin antibody (Fig. 3a–c). CEPI cells that were incubated in the absence of lactoferrin were weakly stained (Fig. 3a). Cells that were incubated with 0.1 or 0.5 mg/mL lactoferrin were more strongly stained (Fig. 3b,c). Lactoferrin uptake was quantitatively analyzed using the Lactoferrin ELISA Kit (Fig. 3d,e). Lactoferrin uptake into CEPI cells increased as the lactoferrin concentration in the medium increased (Fig. 3d). Lactoferrin uptake also increased as the incubation time increased (Fig. 3e).

## Discussion

Because our previous study showed that Se-lactoferrin was useful for treatment of dry eye^18^, we further investigated the effect of application of Se-lactoferrin for dry eye treatment using other dry eye models. Se-lactoferrin eye drops reduced corneal erosion in both rat and rabbit dry eye models (Fig. 1), similar to the results of our previous study^18^. Diquas eye drops were effective in the short-term rabbit dry eye model; however, they were not effective for the TS exposure rat dry eye model. In the TS exposure dry eye model, oxidative stress participates in the development of corneal erosion^2^. Diquas consists of 3% diquafosol ophthalmic solution, which is a specific stimulator of the P2Y2 receptor^23^,^24^. Diquas is a drug for the treatment of dry eye with a novel mechanism of action involving the stimulation of tear and mucin secretion^24^,^25^. Since Diquas probably does not have anti-oxidative stress and anti-toxicity effects, Diquas did not show efficacy for treatment of dry eye in the TS exposure dry eye model. In the short-term rabbit dry eye model, dry eye symptoms were mainly induced by desiccation of the ocular surface. Diquas eye drops showed efficacy in the short-term dry eye model. It can be said that TS exposure led to corneal oxidative stress-related dry eye.

Since Se-lactoferrin eye drops did not irritate the ocular surface of the dry eye model rats in our previous study^18^, ocular irritation induced by Se-lactoferrin eye drops was assayed in the short-term rabbit dry eye model. No irritation was observed on the ocular surface by treatment with Se-lactoferrin eye drops (Table 2). This suggests that Se-lactoferrin as an ophthalmic solution would not be painful to patients.

Our previous study showed that selenium in Se-lactoferrin was imported into corneal epithelial cells and used to synthesize GPx^18^. In the present study, we showed that selenium in Se-lactoferrin was taken into corneal epithelial cells together with lactoferrin via lactoferrin receptors. Lactoferrin uptake into CEPI cells increased as the lactoferrin concentration in the medium and incubation time increased. These quantitative results suggest that lactoferrin receptors are involved in lactoferrin uptake into CEPI cells.

Lactoferrin uptake experiments were performed by incubating CEP1 cells in medium containing 0.1 mg/mL lactoferrin in immunostaining experiments (Fig. 3a–c), while CEP1 cells were incubated in medium containing 2 μg/mL lactoferrin in ELISA experiments (Fig. 3d,e). Comparing the concentration of lactoferrin used in the two experiments, there was a 100 times difference. This difference was due to the low detection sensitivity of immunostaining experiments compared with ELISA experiments. In fact, lactoferrin could not be detected in immunostaining experiments in CEPI cells incubated in medium containing 10 μg/mL lactoferrin (data not shown).

Effect of Se-lactoferrin on the treatment of dry eye was shown in our previous study using another type of dry eye model by removal of lacrimal glands, which was generally used as a dry eye model^18^. Our previous study showed that 8-OHdG level in corneal epithelium of rats increased by smoking treatment^2^ and removal of lacrimal gland^11^. SeP^11^ and Se-lactoferrin^18^ eye drops suppressed increase of 8-OHdG in cornea.

From the results of our previous^18^ and present studies, Se-lactoferrin possessed efficacy for treatment of dry eye in three different types of dry eye models. In addition, there was no evidence that Se-lactoferrin eye drops irritated the ocular surface. We conclude that Se-lactoferrin is an excellent candidate for treatment of dry eye having a novel action involving suppression of oxidative stress.

## Materials and Methods

### Ethics statement

All animal experiments were approved by the Keio University Institutional Animal Care and Use Committee and performed in Keio University, School of Medicine according with Institutional Guidelines on Animal Experimentation at Keio University.

### Evaluation of the effects of Se-lactoferrin on dry eye using the rat dry eye model

Se-lactoferrin was prepared as described in our previous study^18^. The effect of selenium compounds on dry eye was evaluated by applying Se-lactoferrin eye drops to rats with dry eye caused by exposure to tobacco smoke (TS). Six-week-old, male Sprague-Dawley rats were purchased from CLEA Japan, Inc. (Tokyo, Japan). The TS exposure rat model was prepared as described in our previous studies^2^,^26^. Briefly, the rat was placed in an experimental smoking chamber (60 × 40 × 35 cm) with continuous fresh air ventilation for 3 hours per day for 5 days. Three hundred milliliters of mainstream cigarette smoke prepared from cigarettes containing 14 mg tar (Seven Stars; Japan Tobacco, Tokyo, Japan) was injected into the smoking chamber every 30 min for a total of six times over a 3-hour period per day for 5 days during the exposure period. Exposure to TS resulted in an increase in the area of erosion on the ocular surface, which was represented by the area of fluorescein staining^18^.

In the Se-lactoferrin group, one eye in each dry eye rat was treated with 0.1% Se-lactoferrin eye drops containing 18 μM selenium, and the other eye was treated with phosphate-buffered saline (PBS) (n = 12). Our previous study using lacrimal gland-removed dry eye model showed that 0.1% Se-lactoferrin eye drops were most effective for treatment of dry eye^18^. In the Diquas group, one eye in each dry eye rat was treated with 3% Diquas eye drops, and the other eye was treated with PBS as a control drug group (n = 12). Treatment with eye drops was started on the first day of TS exposure. Five μl of each eye drop was administered 4 times per day for 5 days. On each day, the first eye drop was applied before TS exposure and the subsequent eye drops were applied after TS exposure. The nonsmoking group was not exposed to tobacco smoke and did not receive eye drops for treatment (n = 12). After all eye drop treatments, the corneal fluorescein score which was the degree of corneal erosion, was determined by examining fluorescein-stained photographs of the rat ocular surface under anesthesia^18^,^27^. In nonsmoking rats, the corneas were collected to isolate RNA for real-time RT-PCR, or the bulb of eyes was collected for use in the immunohistochemical study.

### Rabbit short-term dry eye model

The effect of Se-lactoferrin ophthalmic solution on the short-term dry eye model in albino rabbits was investigated according to previously described methods^28^,^29^. Rabbits were anesthetized by subcutaneous administration of ethyl carbamate (2 g/kg), and their eyes were held open with an eyelid speculum to desiccate them at 21 °C and 30% relative humidity. Immediately after open the eyes, 50 μl of PBS, 0.1% Se-lactoferrin eye drops, 3% Diquas, or 0.3% sodium hyaluronate ophthalmic solution (Hyalein^®^ ophthalmic solution 0.3%, Hyalein) was instilled onto the corneal epithelial surface. Undesiccated eyes were not kept open by an eyelid speculum. Three hours later, the rabbits were sacrificed by an overdose of sodium pentobarbital, and the eyes were excised. The corneas were stained with 1% methylene blue solution and subsequently washed with saline. The corneas were isolated, and methylene blue was extracted with acetone/sodium sulfate solution for 20 hrs. Its absorbance was measured at 660 nm in a spectrophotometer.

### Assessment of ocular irritation

Assessment of ocular irritation was performed after having confirmed that Se-lactoferrin did not cause eye irritation by single or repeated administration in a previous rabbits and rats studies. Four rabbits were instilled with 50 μL of 0.1, 0.5, or 1.0% Se-lactoferrin in the right conjunctival sac, and the instillation was repeated 6 times at 0.5-hour intervals. At the end of the 6th instillation, the number of eye blinks over a 1-min period was counted. Sixty minutes after the 6th instillation, symptoms of the cornea, iris, and conjunctiva were assessed by the Draize test (Table 1)^30^.

### Expression of lactoferrin receptor in corneal epithelium

Detection of lactoferrin receptors in corneal epithelium was performed by real-time RT-PCR and immunohistochemical analysis. The human corneal epithelial cell line, CEPI-17-CL4 (CEPI) cells^31^ were cultured to 80–90% confluence in medium (Epilife, Life Technologies Corporation, Carlsbad, CA, USA) supplemented with HCGS (Kurabo Industries Ltd., Osaka, Japan) in a 75 cm^2^ flask. Total RNA was extracted from rat corneas or CEPI cells using TRIzol (Life Technologies Corporation). Reverse transcription (RT) was performed using SuperScript III (Life Technologies Corporation) according to the manufacturer’s protocol. The levels of gene expression of lactoferrin receptors, i.e., LRP1 and intelectin-1, were estimated by TaqMan real-time RT-PCR (ABI PRISM 7500 Sequence Detection Systems, Life Technologies Corporation) according to the manufacturer’s protocol. TaqMan probes and other reagents for TaqMan real-time PCR were purchased from Life Technologies Corporation. All data were analyzed with the ΔΔCt method and the mRNA of glyceraldehyde 3-phosphate dehydrogenase (GAPDH) was used as the internal standard.

The bulb of rat eyes was embedded in Optimal Cutting Temperature Compound (Sakura Finetek Japan Co., Ltd., Tokyo, Japan) and frozen, and cryostat sections were made. Immunohistochemical procedures were performed on frozen sections mounted on glass histological slides as described in previous studies^2^,^32^. Briefly, consecutive 5-μm-thick frozen sections were air dried, fixed in acetone at room temperature for 20 minutes, and rehydrated in PBS. Nonspecific binding was inhibited by incubating the specimens with 10% normal goat serum (Life Technologies Corporation) at room temperature for 30 minutes. The specimens were incubated with optimally diluted anti-LRP1 rabbit antibody (Santa Cruz Biotechnology, Inc., Dallas, TX, USA) at 4 °C overnight, followed by incubation with a peroxidase-conjugated goat anti-rabbit IgG antibody (Histofine Simple Stain Max-PO, Nichirei Biosciences Inc., Tokyo, Japan) at room temperature for 45 minutes. LRP1 was visualized by staining with diaminobenzidine tetrahydrochloride. Staining of nuclei was simultaneously performed with hematoxylin. All steps were followed by three washes with PBS. Tissue sections incubated with normal rabbit IgG instead of the primary antibody served as the controls.

Immunostaining of CEPI cells was performed using chamber slide II (AGC Techno Glass Co., Ltd., Shizuoka, Japan). 5 × 10^4^ CEPI cells were sowed in chamber slide II and incubated for 24 hours. After incubation, CEPI cells were washed and fixed with 10% formalin neutral buffer solution. The subsequent procedure was the same as the procedure for tissue sections.

### Lactoferrin uptake into corneal epithelial cells

Lactoferrin uptake into corneal epithelial cells was estimated using CEPI cells. To visualize lactoferrin uptake, lactoferrin in CEPI cells was immunostained using anti-lactoferrin antibody. Anti-lactoferrin rabbit antibody was purchased from Santa Cruz Biotechnology, Inc. as a primary antibody. CEPI cells were cultured to 80–90% confluence in a 75 cm^2^ flask. The medium was changed to a new medium containing 0, 0.1 or 0.5 mg/mL lactoferrin and incubated for 72 hours. CEPI cells were thoroughly washed by PBS and sowed in a chamber slide II. The subsequent procedure was the same as the procedure described in “Expression of lactoferrin receptor in corneal epithelium”.

To quantify lactoferrin uptake into CEPI cells, lactoferrin in CEPI cells was assayed by enzyme immunoassay (EIA) methods. CEPI cells were cultured to 80–90% confluence in a 75 cm^2^ flask. The medium was changed to a new medium containing lactoferrin and CEPI cells were incubated. After thorough washing by PBS, proteins were extracted from the CEPI cells using a homogenizer. Lactoferrin in cellular extracts was measured using the Human Lactoferrin ELISA Kit (Bethyl Laboratories, Inc., Montgomery, TX, USA). Absorbance at 450 nm was measured by a multilabel reader (ARVO SX; PerkinElmer Japan Co., Ltd., Yokohama, Kanagawa, Japan).

## Additional Information

**How to cite this article**: Higuchi, A. *et al*. Selenium-binding lactoferrin is taken into corneal epithelial cells by a receptor and prevents corneal damage in dry eye model animals. *Sci. Rep*. **6**, 36903; doi: 10.1038/srep36903 (2016).

**Publisher’s note:** Springer Nature remains neutral with regard to jurisdictional claims in published maps and institutional affiliations.

## Figures and Tables

**Table 1:** Ocular irritation caused by Se-lactoferrin eye drops.

Se-lactoferrin concentration (%)	Ocular irritation score	Counts of blinking
0.1	1.0 ± 1.2	0.8 ± 1.0
0.5	1.0 ± 1.2	0.5 ± 0.6
1.0	2.0 ± 1.6	0.8 ± 1.5

The results for ocular irritation score and counts of blinking are shown as the mean ± S.D. of 4 eyes in 4 rabbits. Rabbits were instilled with 50 μL of 0.1, 0.5, or 1.0% Se-lactoferrin in the right conjunctival sac, and the instillation was repeated 6 times at 0.5-hour intervals. At the end of the 6th instillation, the number of eye blinks over a 1-min period was counted. Sixty minutes after the 6th instillation, symptoms of the cornea, iris, and conjunctiva were assessed by the Draize test as follows^30^.

Cornea: Score = A × B × 5 A. Opacity-degree of density (area which is most dense is taken for reading).

0: No opacity, 1: Scattered or diffuse area, details of iris clearly visible, 2: Easily discernible translucent areas, details of iris slightly obscured, 3: Opalescent areas, no details of iris visible, size of pupil barely discernible, 4: Opaque, iris invisible.

B. Area of cornea involved.

1: One quarter (or less) but not zero, 2: Greater than one quarter, but less than half, 3: Greater than half, but less than three-quarters, 4: Greater than three-quarters, up to the whole area.

Iris: Score = A × 5 A. Values.

0: Normal, 1: Folds above normal, congestion, swelling, circumcorneal injection (any one or all of these or combination of any thereof), iris still reacting to light (sluggish reaction is positive), 2: No reaction to light, hemorrhage, gross destruction (any one or all of these).

Conjunctivae: Score = (A + B + C) × 2 A. Redness (refers to palpebral conjunctivae only).

0: Vessels normal, 1: Vessels definitely injected above normal, 2: More diffuse, deeper crimson red, individual vessels not easily discernible, 3: Diffuse beefy red.

B. Chemosis.

0: No swelling, 1: Any swelling above normal (includes nictitating membrane), 2: Obvious swelling with partial eversion of the lids, 3: Swelling with lids about half closed, 4: Swelling with lids about half closed to completely closed.

C. Discharge.

0: No discharge, 1: Any amount different from normal (does not include small amounts observed in inner canthus of normal animals), 2: Discharge with moistening of the lids and hairs just adjacent to the lids, 3: Discharge with moistening of the lids and considerable area around the eye.

The maximum total score is the sum of the scores obtained for the cornea, iris, and conjunctivae.

**Table 2:** Expression of lactoferrin receptor in rat cornea.

	GAPDH	LRP1	Intelectin 1
Ct	18.40 ± 0.39	23.92 ± 0.30	37.02 ± 1.77
ΔCt	—	5.53 ± 0.44	18.62 ± 1.54
Fold Change	—	8752	1

The relative expression level of mRNAs was determined by real-time RT-PCR and was estimated using the ΔCt value. ΔCt value was calculated using Ct values of each receptor and GAPDH. If the ΔCt value is lower, the relative RNA level is higher. Fold change was calculated from the ΔCt value. The expression level of intelectin-1 was defined as 1. Results are expressed as the mean ± S.D. (n = 5).

**Table 3:** Expression of lactoferrin receptor in CEPI cells.

	GAPDH	LRP1	Intelectin 1
Ct	20.08 ± 0.89	23.77 ± 0.80	40.10 ± 0.60
ΔCt	—	3.70 ± 0.50	20.03 ± 0.90
Fold Change	—	82343	1

The relative expression level of mRNAs was determined by real-time RT-PCR and was estimated using the ΔCt value. ΔCt value was calculated using Ct values of each receptor and GAPDH. If the ΔCt value is lower, the relative RNA level is higher. Fold change was calculated from the ΔCt value. The expression level of intelectin-1 was defined as 1. Results are expressed as the mean ± S.D. (n = 4).
